# Two-Metal-Ion Catalysis: Inhibition of DNA Polymerase Activity by a Third Divalent Metal Ion

**DOI:** 10.3389/fmolb.2022.824794

**Published:** 2022-03-01

**Authors:** Jimin Wang, William H. Konigsberg

**Affiliations:** Department of Molecular Biophysics and Biochemistry, Yale University, New Haven, CT, United States

**Keywords:** two-metal-ion catalysis, third inhibitory divalent metal ion, bell shaped pol activity plots, Hill coefficient, Brønsted equation

## Abstract

Almost all DNA polymerases (pols) exhibit bell-shaped activity curves as a function of both pH and Mg^2+^ concentration. The pol activity is reduced when the pH deviates from the optimal value. When the pH is too low the concentration of a deprotonated general base (namely, the attacking 3′-hydroxyl of the 3′ terminal residue of the primer strand) is reduced exponentially. When the pH is too high the concentration of a protonated general acid (i.e., the leaving pyrophosphate group) is reduced. Similarly, the pol activity also decreases when the concentration of the divalent metal ions deviates from its optimal value: when it is too low, the binding of the two catalytic divalent metal ions required for the full activity is incomplete, and when it is too high a third divalent metal ion binds to pyrophosphate, keeping it in the replication complex longer and serving as a substrate for pyrophosphorylysis within the complex. Currently, there is a controversy about the role of the third metal ion which we will address in this review.

## Introduction

**FIGURE 1:**
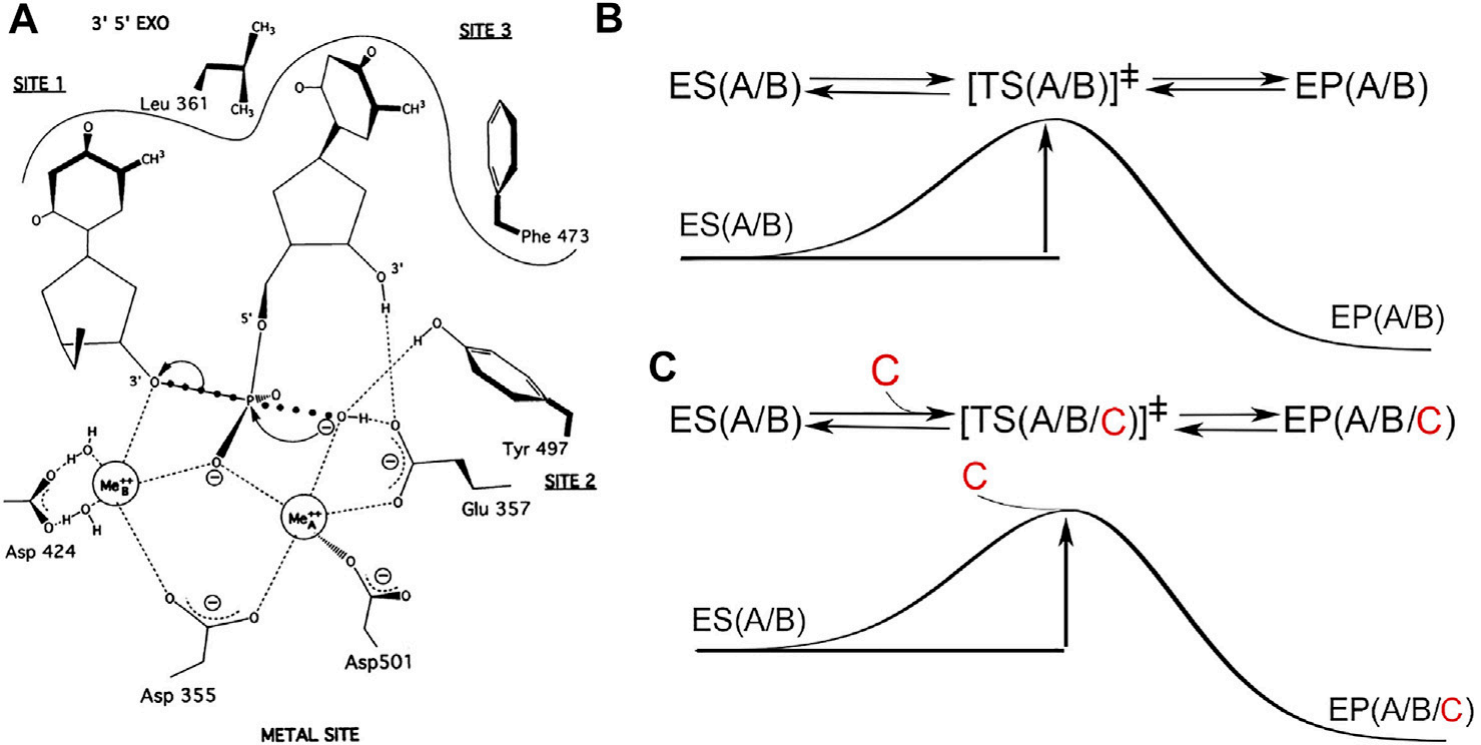
Two-metal-ion catalytic mechanism or three metal ions? **(A)** Hypothetical transition state for the hydrolysis of phosphodiester bond in the 3′,5′-exonuclease active site of Klenow fragment (adapted from Steitz and Steitz, 1993, with permission, ©National Academy of Sciences, 1993). **(B)** Free energy diagram of the classic TS theory involving two metal ions A and B, which are intrisic parts of both ES and EP complexes and remain unchanged during catalysis. Simultaneous bond-breaking and bond-formation at the transition state is a zero order reaction. **(C)** Hypothetical 3-metal ion catalytic mechanism with the third metal ion C entering near or at the TS point, which is not compatabile with the classic TS theory. In this hypothesis, simultaneous bond-breaking and bond formation is a first order reaction, dependent on the occupancy of the third divalent metal ion. This hypothesis predicts a complex dependence of the pol activity on the concentration of divalent metal ions invovling all three sites, which has never been supported by any biochemical data (see text).

The first DNA polymerase (pol) was discovered by Arthur Kornberg and others (Bessman et al., 1958; Lehman et al., 1958), and was shown to be responsible for faithfully copying double-stranded DNA through Watson-Crick basepairing between template and primer strands and between the templating nucleotide and incoming dNTPs. It catalyzed the polymerization reaction with a rate enhancement of over 10^17^-fold relative to the uncatalyzed reaction (Schroeder et al., 2006; Lassila et al., 2011). This unique ability for rate enhancement can be attributed to the stabilization provided by this enzyme specifically only to the transition state (TS) relative to the enzyme-substrate (ES) or enzyme-product (EP) complexes as shown by the steady-state kinetics (Figure 1) (Knowles, 1980; Lassila et al., 2011). The pol-catalyzed reaction absolutely requires divalent Mg^2+^ ions (Goulian et al., 1968; Slater et al., 1972; Kornberg and Baker, 1992). When the logarithm of the steady-state rate was plotted as a function of [Mg^2+^], the slope was +2.0 (not 3) before [Mg^2+^] reached the maximal rate (Bolton et al., 2002). Beyond its optimal concentration, the rate decreased linearly with the increasing concentration, with a slope of -1.0 in both linear and log plots (Slater et al., 1972; Sirover et al., 1979; Vashishtha et al., 2016; Vashishtha and Konigsberg, 2016). The slopes of these plots suggest that pols bind two divalent metal ions for catalytic enhancement and a third divalent metal ion for inhibition according to classic enzymology (Fersht, 2017).

Based on extensive biochemical data, Thomas A. Steitz proposed a two-metal-ion catalytic mechanism for phosphoryltransfer (PT) reactions (Figure 1) (Steitz, 1999). More recently, the discovery that a third divalent metal ion binds to the two products in the pol EP complex (but not to the enzyme itself or to the ES complex) led to the proposal of an alternate mechanism that *assumes* that the third divalent metal ion is directly invovled in catalysis of the chemical step of the pol reaction (Nakamura et al., 2012; Gao and Yang, 2016). We have some concerns about this proposed mechanism and about the underlying assumption because we predict that this third metal ion could also bind the two products outside of an enzyme in solution in the uncatalyzed reaction in the same way as it was observed within the pol complex, without directly interacting with the enzyme itself. If this is the case, this metal ion cannot be assigned to be part of the enzyme-catalyzed rate enhancement in the pol reaction.

The pol active site is formed by several negatively charged residues that serve to bind metal ions. These metal ions and positively charged residues bind triphosphate groups or pyrophosphates and phosphate groups from substrates and products. These charged residues are kept apart from each other so that no direct interaction can occur between them. As a result, substrates and products can readily bind to these charged pol active site residues. Whether simple electrostatic potential (ESP) interactions play an important role in the enzyme-catalyzed PT reactions remains debatable because a similar ESP interaction could be generated by the metal ions outside of an enzyme when bound in a similar manner to the substrates in non-enzymatic reaction (Maegley et al., 1996). However, the ESP of an enzyme active site cannot be simply described by positive or negative charges only. Rather, it is composed of many long-range ESP terms from all charged residues of enzymes nearby in so-called local ESP frustration with very steep gradients (Freiberger et al., 2019). It is likely that these unique ESP gradients help to improve the base selectivity of pols on the basis of small energetic differences in basepairing geometry.

## Two-Metal-Ion Mechanism in Enzyme-Catalyzed Phosphotransfer Reactions

In 1985 Thomas Steitz and others determined the first polymerase structure, namely, the large fragment of *E. coli* pol I or the Klenow fragment (KF) in complex with dTMP bound in the exonuclease active site (Brutlag et al., 1969; Klenow and Henningsen, 1970; Ollis et al., 1985). Following that in 1991 Lorena Beese and Thomas Steitz provided direct evidence for the two-metal-ion catalysis for a PT reaction at the exonuclease active site of the KF (Beese and Steitz, 1991). In 1992 Thomas Steitz and others published the second DNA polymerase structure, HIV-1 RT in complex with an inhibitor, and suggested that all DNA polymerases exhibit a hand-like architecture and have highly conserved carboxylates for binding of two metal ions (Kohlstaedt et al., 1992). In 1993 Thomas and Joan Steitz further extended the requirement for two metal ions that are necessary for catalytic RNA to have a high catalytic function (Figure 1) (Steitz and Steitz, 1993). The two-metal-ion catalytic mechanism has been found to be involved in many known enzymatic processing of nucleic acids (Palermo et al., 2015).

Human/rat pol β and RB69 DNA pol are the three most extensively studied DNA pols using both X-ray crystallography (by Joseph Kraut, Samuel Wilson and others) and biochemistry (by Zucai Suo, Min-Daw Tsai and others), as well as by combined approaches (used by us and others) (Pelletier et al., 1994; Sawaya et al., 1994; Ahn et al., 1997; Wang et al., 1997; Franklin et al., 2001; Batra et al., 2006; Showalter et al., 2006; Xia et al., 2011; Xia et al., 2013; Xia and Konigsberg, 2014; Vyas et al., 2015; Reed et al., 2017; Reed and Suo, 2017; Vyas et al., 2017; Whitaker and Freudenthal, 2020). Other pols that have been extensively studied using both kinetic and structural methods include Dpo4, which binds additional divalent metal ions to the primer/template DNA duplex outside the pol active site (Fiala and Suo, 2004; Irimia et al., 2010). The fact that these metal ions bind makes it very challenging to correlate structures with kinetics, particularly in the chemical step of the pol reaction. In addition to catalysis, divalent metal ions can also modulate noncovalent kinetic behaviors (Dahl et al., 2016).

## The Observation of a Third Metal Ion in the Enzyme-Product Complexes of DNA Polymerases

Wei Yang and others were the first to observe binding of a third divalent metal ion in replication complexes of low-efficiency and low-fidelity lesion-bypass DNA pols (Nakamura et al., 2012; Gao and Yang, 2016). Because the reaction catalyzed by these pols is rather slow, intermediate structures have been characterized by time-resolved X-ray crystallography when crystals were frozen at different times after initiation of the pol reaction (Nakamura et al., 2012; Freudenthal et al., 2013; Gao and Yang, 2016; Jamsen et al., 2017; Reed and Suo, 2017). Yang and others observed a strong correlation between the formation of products and the binding of this third metal ion (Nakamura et al., 2012; Gao and Yang, 2016). In these structures, both ES and EP complexes were simultaneously observed as an inter-convertible mixture at various time points. However, these EP complexes had an extra divalent metal ion bound, which was not found in the other pol complexes discussed above.

One interpretation is that this third metal ion is involved in catalyzing the chemical step in the pol reaction, which would fundamentally change the paradigm of the two-metal-ion catalytic mechanism (Yang et al., 2016; Wu et al., 2017). If, on the other hand, the classic TS theory can fully explain these observations as discussed below, there is no need for revising the original mechanism (Perera et al., 2017; Wu et al., 2017; Yoon and Warshel, 2017; Raper et al., 2018; Stevens and Hammes-Schiffer, 2018). Theoretical calculations can only address whether a given hypothetical chemical mechanism is compatible with chemical principles, but often do not address whether the given mechanism is consistent with existing experimental data. By definition, an enzyme and its cofactors are not reactants or products and not a consumable part of the reaction, thus they remain unchanged in any enzymatic reaction. With this definition, it is hard to classify this third divalent metal ion as a cofactor because it does not directly interact with the enzyme itself but only binds products without an involvement of the enzyme at all (Figure 1).

## Classic View of the Enzyme-Catalyzed Reaction and Its Application to the Presence of a Third Metal Ion

An enzyme does not change the equilibrium of the reaction it catalyzes (Kraut, 1988). It only accelerates the rate of both forward and reverse reactions by the same amount, i.e., it simply lowers the free energy barrier of the reaction. Joseph Kraut noted in 1988 that the classic TS theory originally proposed by Linus Pauling was simple, and yet many investigators who attempted to revise it often misunderstood it, including Kraut himself before 1988 (Pauling, 1946; Pauling, 1948; Kraut, 1988). All elementary reactions must be fully reversible, including the reactions of the polymerization-pyrophosphorylysis pair (Kraut, 1988). The reason DNA pols catalyze DNA synthesis irreversibly *in vivo* is because the pyrophosphate product is continuously degraded by cellular pyrophosphatases and dNTP substrates are continuously resupplied. However, evidence also exists that *E. coli* pol IV and *S. cerevisiea* Rev1 are capable of directly hydrolyzing pyrophosphate, making the polymerization reaction irreversible (Kottur and Nair, 2018; Weaver et al., 2020). The classic TS theory postulates that the TS is a saddle point on the energy landscape with the spatial gradient being zero, i.e., the highest energy point on the reaction trejactory but also the lowest energy point in all other directions with a fixed chemical composition. At the TS, the vibration frequency of an existing bond being broken for formation of a new bond determines the rate of the chemical reaction for either polymerization or pyrophosphorylysis (Kraut, 1988). This property suggests that rate of the chemical reaction at the TS is independent of the TS concentration and is zero order whereas the experimentally observed rate is proportional to the concentration of the TS. Any metal ion must be part of both the ES and EP complexes before it can be considered as part of the TS along the reaction coordinate.

A new metal ion cannot enter the TS saddle point as part of the EP complexes but not as part of the ES complex. If the third metal ion are indeed part of the TS, as hypothesized by some investigators (Stevens and Hammes-Schiffer, 2018), the concentration of the TS would be proportional to the cube of the metal ion concentration when the metal ion concentration is below the saturation point of the tightest bound catalytically metal ion. The logarithm of the pol activity (which is proportional to the concentration of the TS versus the concentration of metal ions) should have a slope of three (Fersht, 2017). If the third metal ion binds only weakly with a much larger dissociation constant than the first two metal ions, the metal ion-dependent activation phase would be biphasic (Fersht, 2017). The logarithm of the pol activity in the first phase has a slope of three when the metal ion concentration is below the saturation point of the first two metal ions. The second phase occurs when the metal ion concentration is below the saturation point of the third metal ion but above that of the first two metal ions. The slope of this new phase is one. Currently, there are no data to support the hypothesis proposed by Stevens and Hammes-Schiffer for any pol.

For pyrophosphorylysis, this third metal ion may be characterized as part of *substrate-*assisted catalysis as it is part of the ES complex (it should be noted that the terms substrate and product are reversed in the forward and reverse reactions) (Figure 1). However, if this third metal ion leaves before the ES complex approaches to the TS (which by definition is at the highest energy point of the reaction coordinate at the saddle point of the energy landscape of the reaction), it cannot contribute to stabilization of the TS. Therefore, it cannot be assigned for any function in pyrophosphorylysis; and if it has no function in pyrophosphorylsis, it cannot have any function in polymerization either, in accordance with the full reversibility principle of any elemental reaction according to the TS theory. Even so, it could still play an indirect role only in the overall reaction of pyrophosphorylysis asymmetrically (but not in polymerization) by retaining the pyrophosphate substrate longer in the ES complex, i.e., increasing the local concentration of the substrate.

When the EP complex of pyrophospholysis is extrapolated from the ES complex, this third metal ion is expected to interact with two phosphate oxygens of Pα of the dNTP product, one with the non-bridging O and the other with the bridging O between the α- and β-phosphate groups. Within an idealized hexacoordination of a Mg^2+^ complex ion (Pavlov et al., 1998), the distance between its two adjacent O ligands is 2.94 Å, which is the distance that the third metal ion is observed in the various pol structures (Nakamura et al., 2012; Gao and Yang, 2016). Within the same Pα phosphate group, the distance between its two O atoms is 2.46 Å (which is shorter by 0.48 Å than the idealized value for simultaneous coordination to any Mg^2+^ ion). If two O atoms of the given phosphate group could become two adjacent ligands for a Mg^2+^ ion with ideal coordination bond length of 2.17 Å, the coordination bond angle O-Mg-O would be 75° (which is a 15° deviation from the ideal value), and if the coordination bond angle to the Mg^2+^ ion would maintain 90°, its coordination bond length would be 1.81 Å (which is a 0.36 Å deviation from the ideal value). With this coordination geometry, other O ligands of Mg^2+^ ion could sterically clash with the O atoms of both α- and β-phosphates of the incoming dNTP. Therefore, on these theoretical grounds, within the energy landscape of the reaction diagram, a Mg^2+^ ion can never be simultaneously coordinate two O atoms of the same phosphate group for stable geometry (particularly if the coordination involves a bridging O atom of reduced charge) as observed in the two products. Because this third Mg^2+^ ion stabilizes only the pyrophosphorylysis ES complex but not the pyrophosphorolysis EP complex, its binding would increase the TS barrier of the reaction. This third metal ion must first leave before pyrophosphorylysis can take place and thus is not part of the catalytic apparatus. The coordination geometry of Mn-O is very similar to Mg-O, although variation of Mn-O coordination bond lengths is larger due to the involvement of its 3*d* electrons compared to that of Mg-O (Sasaki et al., 1979; Pavlov et al., 1998). Due to different rigidities of metal ion coordination octahedra, the two divalent metal ions could have very different degrees of covalency in their coordination bonds.

## Kinetics of Binding of the Third Divalent Ion in Crystal Structures

Does the third divalent metal ion bind to the replication complex immediately before the catalysis of the chemical step of the pol reaction or shortly after it? The kinetic consequences in a crystal differ between these two events (Wang and Smithline, 2019). If the metal ion binds before catalysis, the concentration or the occupancy of this metal ion in the crystals will be larger than the fraction of the formed product. This is especially true if the time is extrapolated to *t* = 0. If it binds after catalysis, the situation is reversed when *t* = 0. With increasing time, the situation becomes more complex because the diffusion rates of this third divalent metal ion and the pyrophosphate product away from the replication complex could vary with time.

**FIGURE 2:**
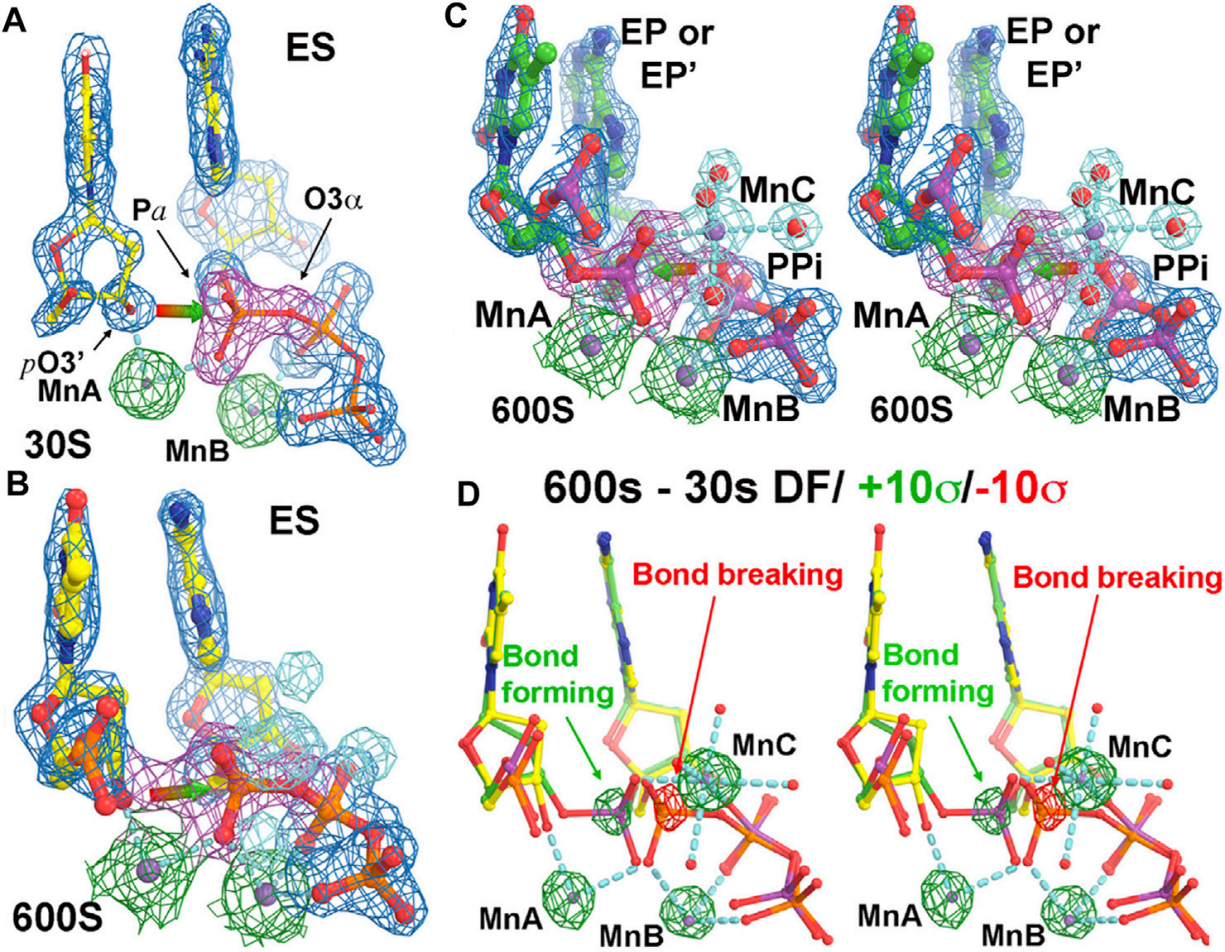
Time-dependent human polymerase η structures (adapted from Wang and Smithline, 2019). **(A)** At 30 s time point. The σ_A_-weighted 2F_o_-F_c_ electron density map contoured at 2σ (blue for overall structure, green for two metal ions A and B, and magenta for the phosphate group). **(B)** At 600 s. New features for MnC and its ligand water molecules (cyan) gradually appear with time. **(C)** Stereodiagram of the 600 s structure. **(D)** Stereodiagram of difference Fourier maps between the 600 s and 30 s structures contoured at +10σ (green) and −10σ (red).

**FIGURE 3:**
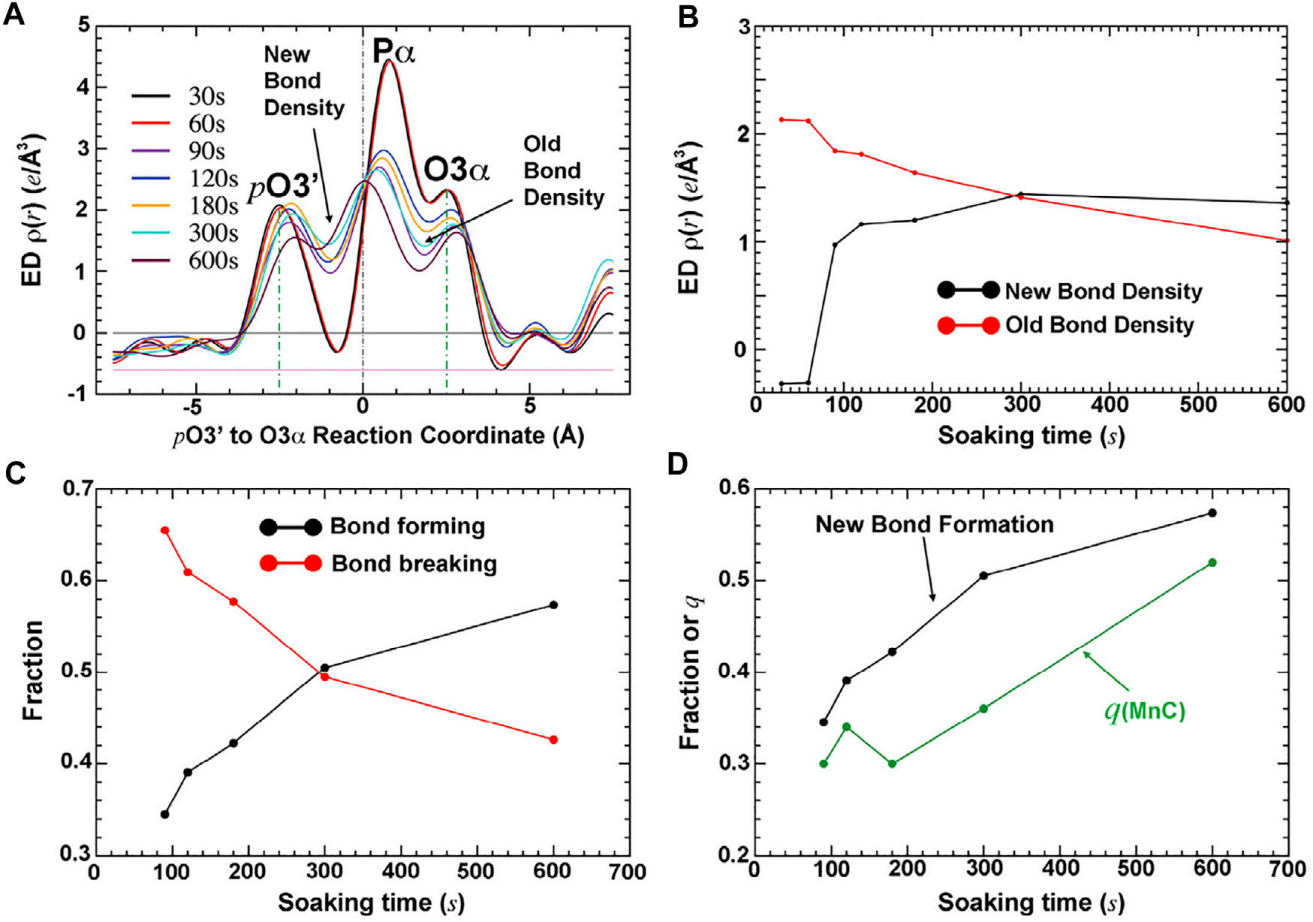
Quantitative analysis of time-dependent electron density for bond formation, bond breaking, and occupancy of the third metal ion MnC (adapted from Wang and Smithline, 2019). **(A)** Plots of the σ_A_-weighted F_o_-F_c_ electron density along the reaction coordinate from *pt* O 3′ to Pα to O3α. **(B)** Electron density at the positions defining bond formation and bond breaking. **(C)** Fractions of bond formation and bond breaking. **(D)** Comparison of new bond formation relative to the occupancy of bound MnC helps to establish the relative order of the first bond formation, followed by binding of MnC.

The fraction of the newly formed bond between the primer-terminal O3′ and Pα of the triphosphate and that of the bond breaking between the Pα and Pβ phosphates of the triphosphate in the complexes can be directly evaluated by the electron density at the midpoint of the corresponding atoms in each pair of the two bonds (Figure 2) (Wang and Smithline, 2019). The relative occupancy of the third metal ion relative to the first two metal ions can be quantitatively estimated using analytic procedures (Figure 3) (Wang and Smithline, 2019). This analysis shows that the occupancy of this third divalent metal ion is always smaller than that of the product formation at any time point, including when the time is extrapolated to the *t* = 0 point. This result suggests that the third metal ion is not involved directly in catalysis of the chemical step of the pol reaction.

## Universal Bell-Shaped Pol Activity Curves as a Function of Divalent Metal Ions and pH

**FIGURE 4:**
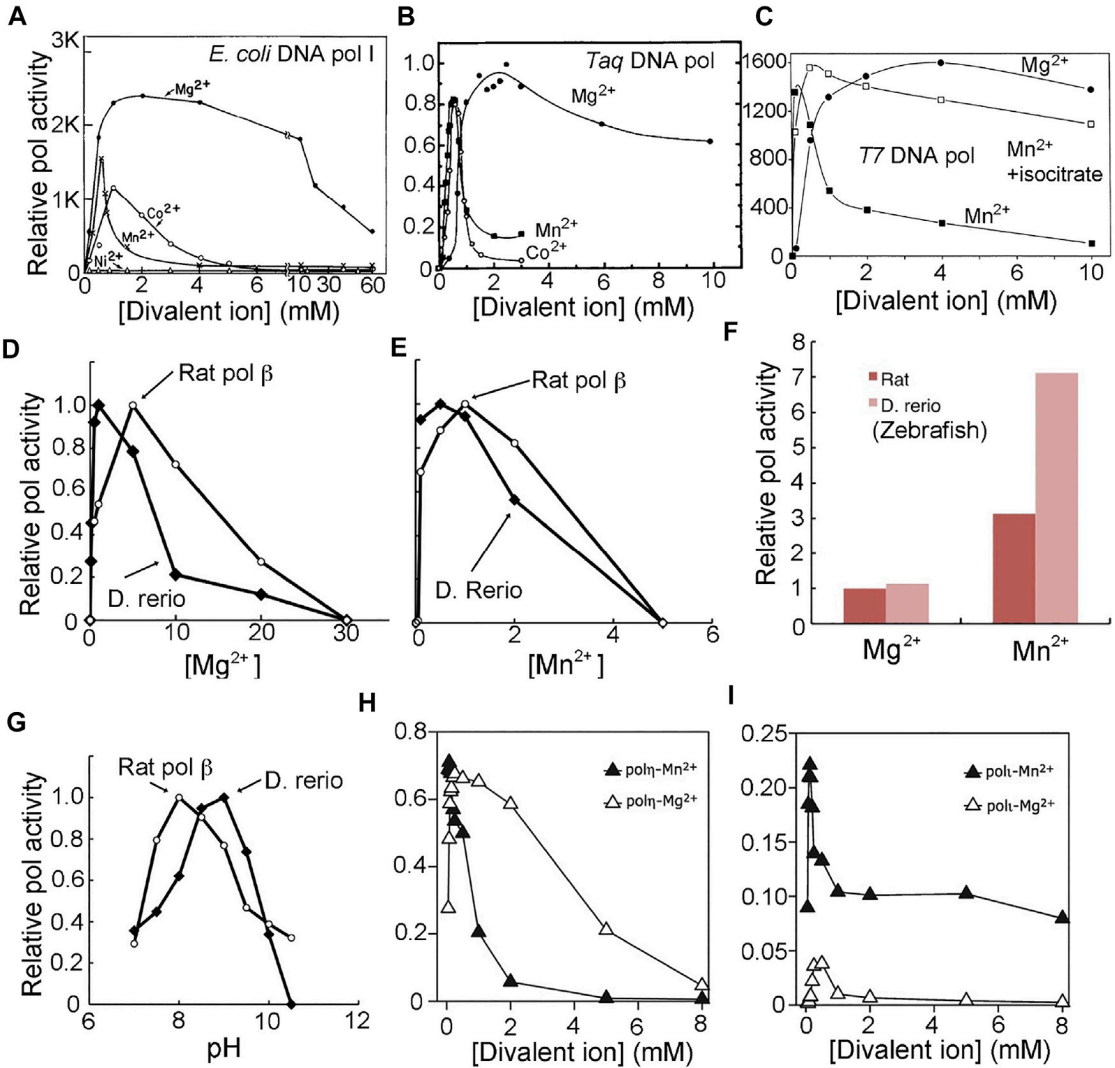
Bell-shaped polymerase activity profiles as a function of divalent metal ions and pH. With the exception of **(A)** and **(C)**, which show nucleotide incorporation under given experimental conditions on the pol product scale, all others are shown on a relative scale. With the exception of **(G)**, which is a pH-activity profile, all others are metal ion-activity profiles. **(A)** *E. coli* DNA polymerase I (adapted from Sirover et al., 1979, J. Biol. Chem.). **(B)** Taq DNA polymerase (adapted from Lawyer et al., 1993, PCR Methods Appl.). **(C)** T7 DNA polymerase (adapted from Tabor and Richardson, 1989, with permission, ©National Academy of Sciences, 1989). **(D–G)** *D. rerio* (zebrafish) pol β and rat pol β (adapted from Ishido et al., 2011, Microbial Cell Factories). **(F)** scale factor between two metal ions and two species. **(H, I)** Human pol η and pol ι (adapted from Frank and Woodgate, 2007, J. Biol. Chem.).

Min-Daw Tsai noted that many kinetic data generated from his laboratory on pol β could be fully explained using the two-metal-ion catalytic mechanism previously known at the time, but could be equally well explained using the new three metal ion-based catalytic mechanism, i.e., his data cannot distinguish between the two mechanisms (Tsai, 2019; Wang and Smithline, 2019). He suggested that new kinetic experiments might be needed to distinguish these two mechanisms. However, extensive kinetic data already exist in the literature that allow one to discriminate between them, even before the classic two-metal-ion catalytic mechanism was proposed. These results are briefly reviewed below (Figure 4), which include data from both pol β and pol η that strongly support the two-metal-ion catalytic mechanism but cannot be explained by a three-metal-ion mechanism.

During the initial partial purification of *E. coli* DNA pol I, it was found that its pol activity absolutely required divalent metal ions and was pH dependent (Goulian et al., 1968; Slater et al., 1972; Kornberg and Baker, 1992). When the pH deviated from its optimal value or when the concentration of divalent metal ions deviated from their optimal value, the pol activity was reduced. Thus the bell-shaped pol activity profile as a function of pH remains universal, and is a hallmark of general acid-general base catalysis. If one acid is involved in catalysis, the overall activity is linearly proportional to the concentration of the acid, which would be exponentially reduced with increasing pH. The slope of the log of activity versus pH plot is based on the Brønsted equation (Brønsted and Pedersen, 1924; Dissanayake et al., 2015). If one base is involved, the overall activity is proportional to the concentration of the base, which would decrease with decreasing pH. The bell-shaped pol activity curve as a function of pH for DNA pols suggests that both acid(s) and base(s) are involved in catalysis. The slope of the plot based on the Brønsted equation shows that there is one acid and one base involved in catalysis, which is consistent with hydrogen/dueterium (H/D) effects of the pol reaction catalyzed by DNA pols (Castro et al., 2007; Castro et al., 2009).

Similarly, a bell-shaped pol activity curve as a function of divalent metal ion concentration for DNA polymerases indicates that divalent metal ions act both as an activation cofactor and as an inhibitor in the overall pol reaction (Figure 4). The semi-log activity versus log[Mg^2+^] or log[Mn^2+^] plot, known as Hill-Langmuir plot, provides the cooperativity Hill coefficients for activation and inhibition of the pol activity, where the coefficient is 2 for the activation phase (Bolton et al., 2002). The optimal metal ion concentration for pol activity is pH dependent, and the optimal pH is dependent on the metal ion concentration because the binding of metal ion can alter the *pKa* value of the general base.

The DNA pol from bacteriophage T4 exhibits pol activity profiles that follow bell-shaped curves for both pH and divalent metal ions (Goulian et al., 1968). In the most recent studies on a DNA pol from a closely-related bacteriophage, RB69, Konigsberg and others showed that the pol activity was reduced by ∼ 2-fold when [Mn^2+^] increased from 10 to 20 mM with the estimated slope of −1 in the plot of log activity versus log[Mn^2+^] (Vashishtha and Konigsberg, 2016; Vashishtha et al., 2016). The optimal activity of *E. coli* DNA pol I is at 0.07 mM [Mn^2+^], and was reduced by more than 3-fold when [Mn^2+^] increased to 0.21 mM (3-fold), highlighting the inhibitory effect of Mn^2+^ ion on its pol activity (Figure 4A) (Slater et al., 1972). The estimated slope of the log activity vs log[Mn^2+^] plot is −1.0 unit, indicating that there is just a single inhibitory binding site. At all pHs, the pol activity on the inhibitory side of the profiles is reduced by approximately 2-fold for every 2-fold increase of divalent metal ion concentration for all DNA pols (Figure 4), implying that there is only a single inhibitory divalent metal ion-binding site according to the Hill-Langmuir plot of the log activity-versus-log concentration for divalent metal ions.

Ekaterina Frank and Roger Woodgate have determined the activity profile of human pol η as a function of both Mn^2+^ and Mg^2+^ ions (Figure 4H) (Frank and Woodgate, 2007). With Mn^2+^, the optimal condition is << 0.1 mM in solution. At ∼1 mM [Mn^2+^], the activity is reduced by 3-fold relative to the optimal [Mn^2+^] condition, and at ∼2 mM [Mn^2+^], the activity is reduced by 10-fold. Gao and Yang have determined that the binding affinity of MnA and that of MnB in crystal is <0.5 mM, and that of MnC is about 3.2 ± 1.5 mM (Gao and Yang, 2016). Subtle differences in kinetic parameters between solutions and crystals is expected because, in crystals, slower diffusion rates can play a role. The MnC site, with weaker affinity in crystals, likely corresponds to an inhibitory site according to the pol activity profile discussed above (Figure 4H). The bell-shaped activity profiles as a function of pH can be readily explained by the deprotonation and protonation processes associated with catalysis. For example, from the maximal activity of rat or zebrafish pol β, the value of log activity versus pH (i.e., −log [H^+^]) decreases with increasing or decreasing pH, and the slope of the resulting lines has a unit value (Figure 4G) (Ishido et al., 2011).

The bell-shaped pol activity profiles as a function of both pH and divalent metal ions have universally been observed for all DNA polymerases (Kornberg and Baker, 1992). This feature has also been described for human/rat DNA pol α, β, γ, λ, and η, for Taq DNA pol, bacterial and archael DNA pol Dpo4, for T7 DNA pol, T4, and RB69 pol, and for avian myeloblastosis viral DNA pol as well as for Ty1 and HIV-1 RT (Figure 4) (Slater et al., 1972; Dube and Loeb, 1975; Sirover et al., 1979; Starnes and Cheng, 1989; Tabor and Richardson, 1989; Lawyer et al., 1993; Bolton et al., 2002; Kokoska et al., 2002; Fiala and Suo, 2004; Castro et al., 2007; Frank and Woodgate, 2007; Ishido et al., 2011; Vashishtha et al., 2016; Vashishtha and Konigsberg, 2016).

## Kinetic Assignment of the Third Divalent Metal Ion as an Inhibitor of the Pol Activity

Linda Rehra-Kranz and others have studied the Mn^2+^-dependent inhibition of the pyrophosphorylysis reaction by the L412M mutant T4 DNA pol using externally supplied pyrophosphate (PPi) (Reha-Krantz et al., 2014). They showed that a high concentration of Mn^2+^ ion clearly inhibited pyrophosphorylysis within the ranges of both the [Mn^2+^] and [PPi] that they studied. However, the nature of the inhibition could not be easily described using classic kinetic schemes because there was a significant amount of the Mn^2+^-PPi complex formation, and because Mn^2+^ ion was both an activator and an inhibitor. The half maximal inhibitory concentration (IC_50_) of Mn^2+^ ion was >3 mM when [PPi] = 4 mM, and IC_50_([Mn^2+^]) was ∼2 mM when [PPi] = 2 mM, and IC_50_([Mn^2+^]) was ∼1 mM when [PPi] = 1 mM, and so on. However, these IC_50_ values are not very meaningful because they there was no report on the activation phase of the reaction, nor the maximal activity at the optimal [Mn^2+^]. Upon comparison with the typical pol activity curves as a function of [Mn^2+^] for DNA pols (Figure 4), the [Mn^2+^] used in their studies was clearly on the inhibitory side of the curve. Thus, the inhibitory Mn^2+^ ion most likely corresponds to the binding at the third Mn^2+^ site.

Given that the third Mn^2+^ site does not bind to the ES complex of the polymerization reaction but binds only to a non-productive ES complex for pyrophosphorylysis, its apparent inhibition with respect to the pol activity profile of the steady-state reaction is only indirect. We propose that this third Mn^2+^ site helps to retain the pyrophosphate product longer than usual within the ternary complex, thus increasing its local concentration and making pyrophosphorylysis increasingly likely. Our proposal is also based the fact that the unreleased pyrophosphate product is the only source of substrate for pyrophosphorylysis. Consistent with our proposal is the observation that when citrate or isocitrate is included in the Mn^2+^ ion-containing reaction, the third Mn^2+^ ion, which has relatively low affinity, can be removed so that the Mn^2+^-dependent inhibition can be eliminated and the maximal pol activity can be restored (Figure 4C) (Tabor and Richardson, 1989).

## Concluding Remarks

The existing biochemical and structural literature on DNA pols is fully consistent with the generalized mechanism of the two-metal-ion catalysis proposed by Thomas Steitz together with the existing TS theory for enzymatic reactions. This review provides a structural basis for the bell-shaped activity profiles of DNA pols as a function of pH and divalent metal ion concentrations. Weak binding of a third divalent metal ion appears to be responsible for retaining pyrophosphate, allowing extra time for pyrophosphorylysis. This could be important for hydrolysis of incorrectly incorporated nucleotide residues for some DNA pols before mismatches are transferred to the exonuclease site.
